# NSAIDs as a Drug Repurposing Strategy for Biofilm Control

**DOI:** 10.3390/antibiotics9090591

**Published:** 2020-09-10

**Authors:** Cláudia Leão, Anabela Borges, Manuel Simões

**Affiliations:** 1 LEPABE—Laboratory for Process Engineering, Environment, Biotechnology and Energy, Faculty of Engineering, University of Porto, Rua Dr. Roberto Frias, s/n, 4200-465 Porto, Portugal; up201607739@fe.up.pt (C.L.); apborges@fe.up.pt (A.B.); 2 DEQ—Department of Chemical Engineering, Faculty of Engineering, University of Porto, Rua Dr. Roberto Frias, s/n, 4200-465 Porto, Portugal

**Keywords:** antibiotic resistance, biofilm eradication, drug repurposing, *Escherichia coli*, NSAIDs, *Staphylococcus aureus*

## Abstract

Persistent infections, usually associated with biofilm-producing bacteria, are challenging for both medical and scientific communities. The potential interest in drug repurposing for biofilm control is growing due to both disinvestment in antibiotic R&D and reduced efficacy of the available panel of antibiotics. In the present study, the antibacterial and antibiofilm activities of four non-steroidal anti-inflammatory drugs (NSAIDs), piroxicam (PXC), diclofenac sodium (DCF), acetylsalicylic acid (ASA) and naproxen sodium (NPX) were evaluated against *Escherichia coli* and *Staphylococcus aureus*. The minimum inhibitory/bactericidal concentrations (MICs and MBCs) and the dose–response curves from exposure to the selected NSAIDs were determined. MICs were found for PXC (800 μg/mL) and ASA (1750 μg/mL) against *E. coli*, and for DCF (2000 μg/mL) and ASA (2000 μg/mL) against *S. aureus*. No MBCs were found (>2000 μg/mL). The potential of NSAIDs to eradicate preformed biofilms was characterized in terms of biofilm mass, metabolic activity and cell culturability. Additionally, the NSAIDs were tested in combination with kanamycin (KAN) and tetracycline (TET). ASA, DCF and PXC promoted significant reductions in metabolic activity and culturability. However, only PXC promoted biofilm mass removal. Additive interactions were obtained for most of the combinations between NSAIDs and KAN or TET. In general, NSAIDs appear to be a promising strategy to control biofilms as they demonstrated to be more effective than conventional antibiotics.

## 1. Introduction

In the past decades, multidrug-resistant (MDR) bacteria have emerged remarkably, causing persistent and severe infections that are extremely difficult to treat with conventional therapies [1,2]. In addition, MDR bacteria may form biofilms, which increase their resistance both to host immune defenses and antibiotics [3,4]. Diverse characteristics of biofilms confer antimicrobial resistance. In particular, the production of a matrix composed by extracellular polymeric substances (EPS) that reduce the diffusion and the penetration of antimicrobial drugs, in addition to protecting the microorganisms from external environmental stresses, like starvation and dehydration [5]. Moreover, the EPS matrix prevents the inner zones of the biofilm from having an efficient intake of oxygen and nutrients, inducing a vegetative state and metabolic inactivity in some cells, named persisters, which become unreachable by antimicrobial agents [6,7]. Antimicrobial resistance is also dictated by the expression of resistance genes that cause the appearance of enzymes that neutralize or degrade antibiotics [8]; the ability to overexpress efflux pumps that allow extrusion of antimicrobial agents from inside the cells [9]; and the ability for apoptosis allowing the community to reestablish itself with the lysed-based nutrients [9]. Moreover, intercellular signaling, a system known as quorum sensing, is important to regulate cellular functions, including virulence and antimicrobial resistance [7,10].

*Escherichia coli* and *Staphylococcus aureus* are commensal microorganisms associated with infections in the community and hospitals. In fact, *E. coli* and *S. aureus* are biofilm-producing bacteria implicated in some of the most common bacterial infections worldwide [3,11,12]. These bacteria develop resistance to several antibiotics through plasmids and the acquisition of gene clusters [13]. Antibiotic resistance has become a major concern as the current antibiotics have limited effectiveness, and the discovery and development of new antibiotics has been declining in recent years [14,15,16]. As of 31 December 2019, data from Dheman et al. [17] show that only 25 systemic antibacterial drugs are under development—17 belonging to established drug classes and 8 from potential new classes. Moreover, most of these drugs are in early stages of development (phase I or II). In addition, the development of new drugs requires large monetary resources and is a very time-consuming process, with a high risk of failing in one of the testing phases [18,19]. Park [20] foresees a total development time of 13–15 years and a cost between USD 2 and 3 billion. Therefore, it is important to find new strategies for the effective treatment of bacterial infections [13,21,22].

Repurposing drugs for new treatment purposes seems to be a promising strategy to overcome the scarcity of effective drugs and the risks associated with new development plans [23,24]. In fact, preclinical, pharmacokinetic, pharmacodynamic and toxicity profiles and the side effects of these drugs are well known, which facilitates other therapeutic applications [19,25]. This pre-existing data allow skipping extensive phase I safety studies and starting clinical trials at phase IIa, reducing substantially the development time and cost [26]. In recent years, some studies acknowledged the antimicrobial effects of several classes of non-antibiotic drugs, more precisely, the nonsteroidal anti-inflammatory drugs (NSAIDs) [27,28,29,30,31,32]. NSAIDs are a class of drugs with analgesic, anti-inflammatory and anti-pyretic properties. These drugs are often used to treat arthritic symptoms and other painful conditions caused by musculoskeletal disorders and trauma events [33,34]. NSAIDs have previously demonstrated antibacterial activity against a wide range of Gram-negative and Gram-positive bacteria [27,28,29]. Their ability to inhibit biofilm formation has also been studied; however, the ability of these drugs to eradicate preformed biofilms has yet to be explored. The antibacterial mode of action of NSAIDs may be related to their ability to affect the integrity of the cytoplasmic membrane of bacteria. In fact, cell permeation to propidium iodide, the release of intracellular *K^+^* and changes in the physicochemical properties of the bacterial surface have been reported—suggesting cytoplasmic membrane damage [32]. Other aspects of their mode of action may be related to the inhibition of DNA synthesis, preventing DNA replication and the repair of the bacterial membrane [35,36,37].

In this work, the antibacterial activity of four NSAIDs, diclofenac sodium (DCF), piroxicam (PXC), acetylsalicylic acid (ASA) and naproxen sodium (NPX) was evaluated against strains of *E. coli* and *S. aureus*. The antibacterial activity of these NSAIDs as function of their concentration and exposure time was also assessed in this study as well as their antibiofilm activity. NSAIDs are drugs often prescribed together with antibiotics as they help to reduce pain and fever associated with bacterial infections [38]. Thus, the ability of these NSAIDs combined with antibiotics to eradicate preformed biofilms was also tested in order to evaluate their ability to potentiate the putative antibiofilm activity of antibiotics. Thus, four broad-spectrum antibiotics from three different classes were selected: ciprofloxacin (CIP; quinolones), tetracycline (TET; tetracyclines) and streptomycin/kanamycin (STR and KAN; aminoglycosides).

## 2. Results and Discussion

### 2.1. Minimum Inhibitory and Bactericidal Concentrations of Selected NSAIDs and Antibiotics

Minimum inhibitory (MIC) and bactericidal (MBC) concentrations were assessed against planktonic *E. coli* CECT 434 and *S. aureus* CECT 976 (Table 1). PXC presented MIC of 800 μg/mL against *E. coli* but did not show bactericidal effects in the range of concentrations tested (0–2000 μg/mL). Contrarily, DCF had no antibacterial activity against *E. coli* in the range of concentrations tested. However, an MIC of 2000 μg/mL against *S. aureus* was found. ASA was the only NSAID with growth inhibitory effects against both bacteria. ASA had MIC of 1750 μg/mL and 2000 μg/mL against *E. coli* and *S. aureus*, respectively. NPX caused no antibacterial effects against both bacteria in the range of concentrations tested. None of the NSAIDs showed bactericidal activity for any of the bacteria in the range of concentrations tested. To sum up, PXC only presented inhibitory activity against *E. coli*, while DCF only had inhibitory activity against *S. aureus*, and ASA presented inhibitory activity against both bacteria. Thus, these were the NSAIDs selected for further experiments.

Due to the hydrophobic properties of PXC, some dissolution problems in Mueller–Hinton (MH) broth were detected. A precipitate is typically formed, preventing the action of the solubilized fraction of the drug, which culminates in an initial MIC of 2000 μg/mL against *E. coli*. Lactose was added to MH broth in order to avoid precipitate formation. However, after a few hours, some precipitation was visible for high PXC concentrations. In addition of being a common excipient used for solutions with PXC, lactose has a high availability and a high cost-benefit ratio, which is why it is often used in pharmaceutical formulations [39,40,41].

A study by Chan et al. [28] provided significant evidences on the antimicrobial effects of ASA and DCF against a wide number of Gram-negative (*E. coli* ATCC 25922, *Klebsiella pneumoniae* ATCC 10031, *Pseudomonas aeruginosa* ATCC 10145, *Enterobacteraerogenes* and *Salmonella choleraesuis* ATCC 10708) and Gram-positive bacteria (*Bacillus cereus* ATCC 14579, methicillin-sensitive *S. aureus*—MSSA-ATCC 25923, methicillin-resistant *S. aureus*—MRSA-ATCC 33591 and two MRSA clinical strains). In fact, they suggested that ASA displayed a broad-spectrum of antibacterial activity against species of Gram-positive and Gram-negative bacteria. On the other hand, DCF only showed antibacterial activity against Gram-positive bacteria [28]. These authors reported MIC for DCF against *S. aureus* (1250 μg/mL) and *E. coli* (5000 μg/mL) [28]. The results found in that study corroborate the present results, proposing that *S. aureus* is more susceptible to DCF than *E. coli*. Regarding ASA, the same study found MIC of 2500 μg/mL and 5000 μg/mL for *S. aureus* and *E. coli*, respectively. Although the results for *S. aureus* are in agreement with the present study, the MIC for *E. coli* is much higher. This difference is arguably related to the use of different strains in the independent studies. In Abbas et al. [42], MICs between 625 and 1250 μg/mL were found for PXC against several clinical strains of *E. coli*. Regarding NPX, Laudy et al. [43] reported MIC above 3200 μg/mL for three different *E. coli* strains (*E. coli* ATCC 25922, *E*. *coli* NCTC 10538 and *E*. *coli* NCTC 8196), corroborating the present results for this specific NSAID.

The antibacterial activity of four selected broad-spectrum antibiotics, CIP, TET, STR and KAN, was tested against *E. coli* and *S. aureus* by the disc diffusion method in solid medium, and the inhibition zone diameters (IZD) were measured (Table 2). The results showed that TET and KAN were the two antibiotics with lowest IZD, meaning that *E. coli* and *S. aureus* were more resistant to TET and KAN than for CIP and STR. Based on the interest for assessing the selected NSAIDs in the modification of bacterial resistance, the two least effective antibiotics were selected for further studies. In general, *S. aureus* seems to be more susceptible to the selected antibiotics than *E. coli*, as the IZD values were higher for CIP, TET and STR; however, the differences were not statistically significant (*p* > 0.05). KAN had comparable effects against both bacteria (*p* < 0.05).

IZDs of 26 mm for KAN, valid for both species, and of 25 and 30 mm for TET against *E. coli* and *S. aureus*, respectively, were reported in previous studies [9,44]. Regarding CIP, maximum of 40 and 30 mm for *E. coli* and *S. aureus* were described [44]. The IZD values determined for the selected antibiotics and strains are slightly higher than those found in the literature, as a consequence of the use of distinct strains [44,45]. However, comparable values were found for KAN [44,45]. Moreover, *E. coli* was found to be more resistant to TET than *S. aureus* [9,44,45]. For further combinatorial assays between NSAIDs and antibiotics, the MIC and MBC for KAN and TET against *E. coli* and *S. aureus* were determined (Table 3). KAN presented MIC of 24 and 3 μg/mL and TET exhibited MIC of 6 and 4 μg/mL for *E. coli* and *S. aureus*, respectively. Therefore, *E. coli* is the most resistant bacterium to the selected antibiotics, however, the differences were not statistically significant (*p* > 0.05). In addition, *E. coli* was more susceptible to TET, while *S. aureus* was more susceptible to KAN. MBC and MIC of KAN against *E. coli* were found to be equivalent.

According to the Clinical & Laboratory Standards Institute (CLSI) guidelines and the quality control ranges, MIC for KAN should be between 1 and 4 µg/mL for both bacteria [44]. In fact, the values obtained for KAN against *S. aureus* were within this range; however, *E. coli* seems to be more resistant than the control strains of the CLSI-MIC values out of the range described [44]. Regarding TET, MIC must range between 0.12 and 1 µg/mL for *E. coli* and between 0.5 and 2 µg/mL for *S. aureus*. The values obtained in this work were slightly higher than the values of the CLSI, an effect arguably related to the bacterial strains used [44].

### 2.2. Effect of Different Doses of the Selected NSAIDs on Culturability of E. coli and S. aureus Planktonic Cells

**Figure 1:**
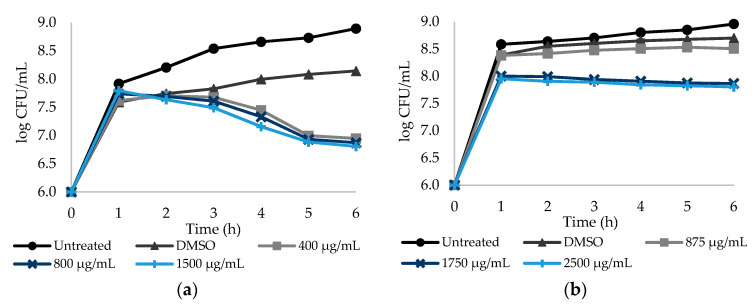
Dose–response curves. The *y*-axis reports the log CFU/mL of *E. coli* planktonic cells, while the *x*-axis reports the period of 6 h of exposure to the NSAIDs: (**a**) PXC (piroxicam) at three different concentrations (1/2× MIC, MIC and a concentration above the MIC), grown in broth culture medium with lactose and treated with DMSO; (**b**) ASA (acetylsalicylic acid) at three different concentrations (1/2× MIC, MIC and a concentration above the MIC), treated with DMSO. Cells without NSAIDs and cells with DMSO were used as negative controls. Mean values ± SD are presented.

In order to assess the relationship between the culturability of *E. coli* and *S. aureus* cells and NSAID concentration, the number of colony forming units (CFU) was determined over time. Dose–response curves are provided for a 6 h period, taking into account that no significant differences (*p* > 0.05) were found after 6 h (until 24 h—data not shown). All the NSAID were tested at three different concentrations, corresponding to a sub-MIC, the MIC and a concentration above MIC. Figure 1a,b show the log CFU/mL of *E. coli* after exposure to PXC and ASA during 6 h. PXC was tested at 400, 800 and 1500 µg/mL, while ASA was tested at 875, 1750 and 2500 µg/mL.

PXC had a maximum of 1.34 log CFU/mL reduction for the highest concentration—1500 µg/mL (Table S1—see Supplementary Data)—when compared to the control with DMSO. PXC at 400 and 800 µg/mL caused 1.20 and 1.27 log CFU/mL reductions, respectively. Figure 1a also shows that the culturability of *E. coli* was not dependent on the PXC concentration, as there are no statistically significant differences between the two concentrations tested (*p* > 0.05). For ASA, the culturability of *E. coli* was dose dependent—a significant difference between the sub-MIC and MIC was found (*p* < 0.05) (Figure 1b). For this NSAID, a maximum reduction of 0.90 log CFU/mL was obtained for the highest concentration tested—2500 µg/mL (Table S1—see Supplementary Data). For 875 and 1750 µg/mL, log CFU/mL reductions of 0.19 and 0.83 were found, respectively.

**Figure 2:**
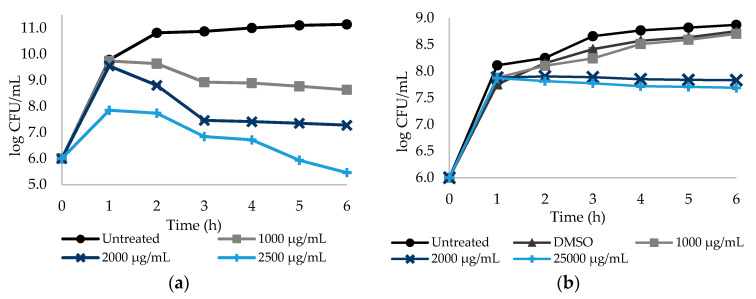
Dose–response curves. The *y*-axis reports the log CFU/mL of *S. aureus* planktonic cells, while the *x*-axis reports the period of 6 h of exposure to the NSAIDs: (**a**) DCF (diclofenac sodium) at three different concentrations (1/2× MIC, MIC and a concentration above the MIC), untreated with DMSO; (**b**) ASA (acetylsalicylic acid) at three different concentrations (1/2× MIC, MIC and a concentration above the MIC) treated with DMSO. Cells without NSAIDs and cells with DMSO were used as negative controls. Mean values ± SD are represented.

Similarly, the effect of DCF and ASA in *S. aureus* culturability reduction was analyzed during 6 h. DCF and ASA were tested at 1000, 2000 and 2500 µg/mL (Figure 2). *S. aureus* culturability was found to be dependent on the DCF concentration (*p* < 0.05). Reductions of 2.50, 3.86 and 5.68 log CFU/mL were found for DCF at 1000, 2000 and 2500 µg/mL, respectively (Figure 2a, Table S1—see Supplementary Data). ASA caused reductions of 0.92 and 1.06 log CFU/mL at 2000 and 2500 µg/mL, respectively (Figure 2b; Table S1—see Supplementary Data). However, there was no log CFU/mL reduction for ASA at 1000 µg/mL (*p* > 0.05). The effect of ASA on log CFU/mL reduction was found to be dependent on the concentration—the two highest concentrations promoted a reduction of circa 1 log CFU/mL, while no reduction was observed from sub-MIC exposure (*p* < 0.05).

Figure 1 and Figure 2 show that DCF seems to be the only NSAID with an effective dose–response effect. This behavior may be related to the NSAIDs’ hydrophobicity and solubility properties. PXC and ASA hold low solubility values of 0.14 and 1.46 mg/mL (values predicted from ALOGPS 2.1 software), respectively, besides the high logP values which suggests high hydrophobicity. From ALOGPS 2.1 software, a logP of 2.20 was predicted for PXC meaning a 158:1 partitioning in organic:aqueous phases, while, for ASA, a logP of 1.43 was predicted, meaning a 27:1 partitioning in organic:aqueous phases. In fact, as stated before, lactose was added to MH broth in order to stabilize the solution with PXC. Although lactose initially stabilized the solution, over the experimental time, a precipitate was formed for higher concentrations of PXC. The same phenomenon may happen with ASA, even though it is not noticeable to the naked eye. This proposes that, although there is an increase in the concentrations of both NSAIDs, the amount of drug in solution is almost the same. In fact, the higher the concentration, the higher the amount of precipitate formed. Contrarily, DCF is substantially less hydrophobic and more water-soluble than PXC and ASA. Studies reported values of logP of 0.7, meaning a 5:1 partitioning in organic:aqueous phases, and a water solubility of 15.9 mg/mL for DCF [46,47].

The antibacterial mode of action of NSAIDs is apparently multifactorial. Oliveira et al. [32] propose that ibuprofen affects the integrity of cytoplasmic membrane, proven by cell permeation to PI, release of intracellular *K^+^* and changes of the physicochemical properties in the bacterial surface. Mazumdar et al. [35] demonstrated the role of DCF in the inhibition of DNA synthesis, in addition to a moderate membrane-damaging action against *Listeria monocytogenes* ATCC 51744. Similarly, several authors proposed the inhibition of DNA synthesis through binding to the DNA polymerase III β subunit as a mode of action of NSAIDs, preventing DNA replication and repair or impairment of membrane activity [26,36,37].

### 2.3. Effect of Selected NSAIDs and Antibiotics on the Control of E. coli and S. aureus Biofilms

**Figure 3:**
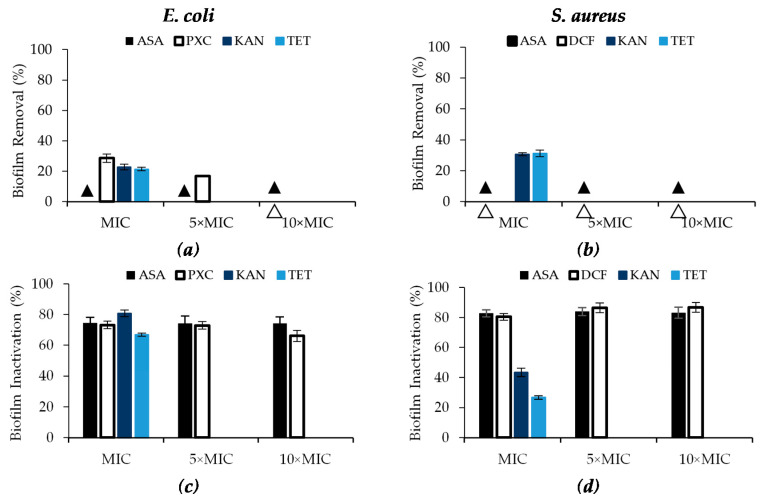
Effects of PXC (piroxicam), DCF (diclofenac sodium), ASA (acetylsalicylic acid), at three different concentrations (MIC, 5× MIC and 10× MIC) and KAN (kanamycin) and TET (tetracycline) at MIC (in this case, 5× MIC and 10× MIC were not tested) against *E. coli* and *S. aureus* after 24 h of exposure in terms of: (**a**,**b**) biomass removal (%) and (**c**,**d**) biofilm inactivation. ▲/∆ represents 0% biofilm removal. Mean values ± SD of three independent experiments are illustrated.

**Figure 4:**
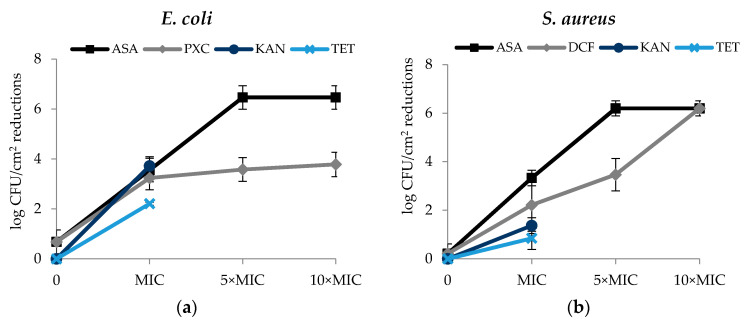
Effect of PXC (piroxicam), DCF (diclofenac sodium), ASA (acetylsalicylic acid), at three different concentrations (MIC, 5× MIC and 10× MIC) and KAN (kanamycin) and TET (tetracycline) at MIC (in this case, 5× MIC and 10× MIC were not tested) in the biofilm culturable cells—log CFU/cm^2^ reduction. (**a**) *E. coli* and (**b**) *S. aureus*, after 24 h exposure. Mean values ± SD of three independent experiments are illustrated.

The effect of PXC, DCF and ASA at three different concentrations (MIC, 5× MIC and 10× MIC), and the effect of KAN and TET at MIC on the control of pre-established *E. coli* and *S. aureus* biofilms (24 h old) were studied through the quantification of the biomass, metabolic inactivation and number of culturable cells (Figure 3 and Figure 4; Tables S2, S3 and S5—see Supplementary Data). Although, putative limitations in NSAIDs’ solubility have been observed in the tests with planktonic cells, with no dose–response effect occurring, biofilms were also exposed to concentrations higher than the MIC. In fact, planktonic cells do not predict the behavior of biofilms. These complex biological structures are highly resistant to conventional antibiotics and extremely difficult to be removed from surfaces, which helps to explain the appearance of some recalcitrant infections [48,49].

Regarding biomass removal, only PXC was able to remove *E. coli* biofilms (Figure 3a). In fact, PXC at MIC removed 28.6% of biomass showing moderate efficacy (Table S5—see Supplementary Data), while 5× MIC removed only 16.8% (low efficacy) and at 10× MIC there was no removal. Biomass removal was found to have an inverse relationship with the concentration (*p* < 0.05). ASA caused no *E. coli* and *S. aureus* biofilm removal as well as DCF for *S. aureus* biofilms (Figure 3a,b). KAN and TET had low efficacy in *E. coli* biofilm removal (22.8% and 21.5%, respectively) and moderate efficacy against *S. aureus* biofilms (30.6% and 31.2%, respectively).

The three NSAIDs showed high efficacy in metabolic inactivation, with reductions between 66.1% and 86.7% (Table S5—see Supplementary Data). Regarding ASA, the percentage of biofilm inactivation was not concentration dependent, as the values were similar for the three concentrations tested (*p* > 0.05). ASA promoted percentages of biofilm inactivation of circa 74.2% for *E. coli* and 83.3% for *S. aureus*. Similarly, the percentage of inactivation of *E. coli* with PXC and *S. aureus* with DCF were not concentration dependent. Inactivation percentages were between 66.1% and 73.2% for PXC, and 80.4% and 86.7% for DCF. Similarly, high efficacies were found for KAN and TET against *E. coli*—biofilm inactivation of 80.8% and 66.8%, respectively (*p* < 0.05). Moderate effects in the inactivation were found from KAN and TET application against *S. aureus* biofilms (43.5% and 26.8%, respectively) (*p* < 0.05).

Regarding the culturability of biofilm cells, in general, the effects of the selected NSAIDs were concentration dependent—reductions of log CFU/cm^2^ increased with the concentrations of the selected NSAIDs. The effect of ASA on the *E. coli* and *S. aureus* biofilm culturable cells was similar (Figure 4a,b). In fact, ASA caused log CFU/cm^2^ reductions of 3.55 (MIC), 6.46 (5× MIC) and 6.46 (10× MIC) for *E. coli* and 3.33 (MIC), 6.19 (5× MIC) and 6.19 (10× MIC) for *S. aureus* (*p* < 0.05). Therefore, total log CFU/cm^2^ reduction of both bacteria was observed for the two highest concentrations tested (5× MIC and 10× MIC). DCF caused total log CFU/cm^2^ reduction (6.19) for *S. aureus* and for the highest concentration tested (10× MIC). Significant log CFU/cm^2^ reductions were also obtained using DCF at the MIC (2.22 log CFU/cm^2^ reduction) and 5 × MIC (3.47 log CFU/cm^2^ reduction) (*p* < 0.05). Interesting results were also observed with PXC at the different concentrations tested, 3.24 log CFU/cm^2^ reduction (MIC), 3.58 log CFU/cm^2^ reduction (5× MIC) and 3.78 log CFU/cm^2^ reduction (10× MIC) (*p <* 0.05). MIC of KAN and TET caused reductions of 3.72 log CFU/cm^2^ and 2.21 log CFU/cm^2^ against *E. coli*, respectively. Reductions of 1.37 log CFU/cm^2^ (KAN) and 0.85 log CFU/cm^2^ (TET) were observed for *S. aureus*.

The present work is focused on the ability of NSAIDs to affect preformed biofilms. Most of the studies analyzed the effects of NSAIDs in the early stages of biofilm formation, particularly the adhesion of planktonic cells. The antibiofilm mode of action of NSAIDs is not totally understood; however, few studies investigated the possible mode of action of NSAIDs in the control of preformed biofilms, which highlights the importance of conducting research in this regard [10,50,51,52,53].

Silva et al. [10] proposed that the NSAIDs’ mode of action may be related to the easy diffusion of these drugs through the biofilm allowing a direct interaction with the pathogen and the disintegration of the biofilm structure. In the same study, Silva et al. [10] proposed that DCF may interfere with the *P. aeruginosa* quorum-sensing system. Abbas [50] studied the ability of DCF to suppress *P. aeruginosa* virulence factors regulated by the quorum-sensing system and observed that several virulence factors were suppressed, including swimming and twitching motilities, biofilm formation and the expression of protease, hemolysin, pyocyanin and pyoverdin. Similarly, El-Mowafy et al. [51] studied the inhibition of *P. aeruginosa* virulence factors by ASA and found effects on the motility and a decreased ability for biofilm formation. In addition, ASA decreased the expression of proteases, elastase and pyocyanin, and the expression of some genes (*lasI*, *lasR*, *rhlI*, *rhlR*, *pqsA* and *pqsR*) and toxins (*exoS* and *exoY*) by *P. aeruginosa*. Cramton et al. [52] further showed evidence that some NSAIDs, such as DCF and meloxicam, regulated negatively the expression of the *icaA* gene, which belongs to the operon *icaADBC*. This operon encodes an important component of the polysaccharides from the extracellular matrix, the polysaccharide intercellular adhesin (PIA), which is the main component of the EPS matrix of *S. aureus*. This is an indicator of the ability of NSAIDs to degrade preformed biofilms [52]. Furthermore, Mahmoud et al. [53] tested several NSAIDs (DCF, PXC, meloxicam and ketoprofen), and concluded that DCF is the NSAID with the highest ability to degrade preformed biofilms of diverse *S. aureus* and *Candida albicans* strains. However, PXC, meloxicam and ketoprofen also showed great ability to degrade preformed biofilms of all the *S. aureus* strains tested. On the other hand, PXC was the only NSAID with no ability to degrade preformed *C. albicans* biofilms [53].

### 2.4. Effect of Dual Combinations of Selected NSAIDs with KAN and TET on the Control of E. coli and S. aureus Biofilms

The selected NSAIDs were combined with KAN and TET and were scored according to the total combinatorial index (∑*C_I_*) values (Table 4). It is important to emphasize that all the NSAIDs were tested at three concentrations, MIC, 5× MIC and 10× MIC, while KAN and TET were tested only at the MIC. In general, most of the interactions were classified as additive. Starting with *E. coli*, PXC had additive interactions both with KAN and TET in the culturability of biofilm cells for all the concentrations tested. Additive interactions were also scored for PXC with TET in terms of biofilm inactivation and for the three concentrations. The same was observed for PXC (10× MIC) with KAN and TET in terms of biomass reduction. PXC (at all the concentrations) presented indifferent interactions with KAN in terms of biofilm inactivation. Regarding biomass reduction, indifferent interactions were also found for PXC (MIC and 5× MIC) with KAN and PXC (5× MIC) with TET. PXC at MIC with TET were scored with an antagonistic interaction. This result exposes the potential hazards from the empirical combination of different drugs, which can reveal an unexpected antagonistic interaction. Thus, it is important to take into account the risks from the application of a combined therapy. Regarding ASA, except for ASA at MIC with KAN, which presented an indifferent interaction in terms of culturability of *E. coli* biofilm cells, all the other interactions of ASA at all the concentrations tested with both antibiotics were scored as additive (for biofilm removal/inactivation and culturability).

Regarding *S. aureus*, the interactions for ASA were exactly the same obtained for ASA against *E. coli*. In fact, only ASA at MIC with KAN caused an indifferent interaction in terms of culturability. Regarding biomass reduction and biofilm inactivation, all the remaining interactions were scored as additive. Finally, for DCF indifferent interactions were obtained at MIC with KAN in terms of biomass reduction. In terms of culturability, indifferent interactions were found at DCF MIC and 5× MIC with KAN and at 5× MIC with TET. The remaining interactions were classified as additive.

The percentages of biomass removal, biofilm inactivation and log CFU/cm^2^ reduction values for the combinations between KAN and TET and the selected NSAIDs are presented in Table S4 (see Supplementary Data). According to the percentages of biomass removal and inactivation, the antibiotics and the antibiotic–NSAID combinations were classified in terms of efficacy and the results are illustrated in Table S5 (see Supplementary Data). Regarding *E. coli*, low efficacies were found in biofilm mass removal from exposure to the combinations of PXC-KAN (MIC—17.8%; 5× MIC—19.0%) and PXC-TET (MIC—2.4%; 5× MIC—16.3% and 10× MIC—20.5%). The combination of 10× MIC PXC with KAN showed moderate efficacy, with 25.2% removal of the *E. coli* biofilm mass. Combinations of ASA-KAN (MIC—30.0% and 5× MIC—30.0%) and ASA-TET (MIC—25.4%) also showed moderate efficacy in biofilm removal. The remaining concentrations showed low efficacy in biofilm removal. Regarding biofilm inactivation, all the combinations using PXC and the selected antibiotics presented high efficacy—percentages ranging from 63.0% and 85.3%. Combinations of ASA and AN (5× MIC—92.6% and 10× MIC—92.5%) and TET (5× MIC—92.7% and 10× MIC—92.7%) showed excellent efficacies. ASA at MIC exhibited biofilm inactivation values of 86.3% and 88.8% for KAN and TET, respectively, representing high efficacy.

Regarding *S. aureus*, the combination between DCF and KAN caused low biofilm removal (MIC—12.2%; 5× MIC—16.8% and 10× MIC—23.5%). A similar behavior was observed for DCF with TET (MIC—16.2% and 5× MIC—22.5%); however, 10 × MIC DCF with TET presented a moderate efficacy (32.4%) in biofilm removal. Dual combinations of ASA with KAN (MIC—31.4% and 5× MIC—28.1%) and TET (MIC—28.4% and 5× MIC—27.4%) also caused moderate efficacy in biofilm removal. ASA at 10× MIC with KAN (16.7%) and TET (22.2%) showed low efficacy in biofilm removal. Therefore, an increase in ASA levels reduced biofilm removal for both bacteria. Regarding biofilm metabolic inactivation, all the combinations between DCF and the selected antibiotics showed high efficacy (KAN: MIC—82.6%, 5× MIC—89.3% and 10× MIC—89.2%; TET: MIC—80.2%, 5× MIC—89.2% and 10× MIC—89.2%). Similar results were obtained for ASA in combination with KAN and TET, as all the combinations showed high efficacy in biofilm metabolic inactivation (KAN: MIC—73.3%, 5× MIC—76.2% and 10× MIC—77.2%; and TET: MIC—74.4%, 5× MIC—76.2% and 10× MIC—76.7%).

The performance of the selected NSAIDs combined with antibiotics in culturability reduction was also assessed, and all combinations between the selected antibiotics and ASA or DCF promoted total log CFU/cm^2^ reductions. Combinations of ASA and KAN, against *E. coli* (MIC—4.04; 5× MIC—6.46 and 10× MIC—6.46) and *S. aureus* (MIC—2.73; 5× MIC—6.19 and 10× MIC—6.19) are the only combinations that promoted total log CFU/cm^2^ reduction for 5× MIC and 10× MIC. All the others showed total log CFU/cm^2^ reduction only for the maximum concentration tested (10× MIC): DCF with KAN (MIC—3.00; 5× MIC—3.15 and 10× MIC—6.19), DCF with TET (MIC—0.99; 5× MIC—3.05 and 10× MIC—6.19), ASA with TET against *E. coli* (MIC—1.35; 5× MIC—3.86 and 10× MIC—6.46) and ASA with TET against *S. aureus* (MIC—1.04; 5× MIC—2.06 and 10× MIC—6.19). As stated before, PXC was the only NSAID that did not promote total log CFU/cm^2^ reduction in combination with the selected antibiotics. For PXC, log CFU/cm^2^ reductions of 2.48, 2.57 and 2.64 with KAN and 1.76, 1.96 and 2.24 with TET were found against *E. coli* at MIC, 5× MIC and 10× MIC, respectively.

ANOVA was performed for the results obtained from ASA exposure (the only compound tested for both bacterial strains), allowing to observe no significant differences between the percentages of biomass and log CFU/cm^2^ reduction for ASA combined with KAN/TET (*p* > 0.05). Moreover, *E. coli* showed to be significantly more susceptible to the combination of ASA and KAN/TET than *S. aureus* (*p* < 0.05).

According to the results and classifications obtained (Table 4 and Tables S2–S4) it is possible to conclude that, in general, no advantages were found from the combination of NSAIDs with the selected antibiotics. In fact, in terms of biofilm inactivation and culturability reduction, for most of the cases, the values for NSAIDs were higher than these for the antibiotics or the combinations.

Even if no remarkable benefits from the antibiotic–NSAID combinations were found in this study, previous reports showed that NSAIDs improved the activity of several antibiotics. Chan et al. [28] studied the effect of the combination of two NSAIDs, ASA and ibuprofen, with two antibiotics, cefuroxime and chloramphenicol, against MRSA and found synergy between ASA–ibuprofen and chloramphenicol–cefuroxime. These authors claimed that ASA and ibuprofen increased the susceptibility of *S. aureus* towards the selected antibiotics [28]. Mazumdar et al. [35] studied the combinatorial antibacterial activity of DCF and STR against *S. aureus*, *E. coli* and mycobacteria and found synergy from DCF–STR combination against the three bacteria. Ahmed et al. [54] investigated the combinatorial effect of four NSAIDs (DCF, ASA, indomethacin and ibuprofen) with five β-lactam antibiotics (ampicillin, amoxicillin, amoxicillin + clavulanic acid, cephalexin and cefotaxim) against *P. aeruginosa* and *K. pneumoniae* strains. Their results showed that all the NSAIDs had synergistic interactions with the selected antibiotics. They also found that DCF caused higher antibacterial effect when combined with aminoglycosides [54]. Ahmed et al. [54] also reported the combined effect of DCF, ASA, indomethacin and ibuprofen with five antibiotics (amoxicillin, augmentin, cefotaxime, ciprofloxacin and gentamicin) against diverse *E. coli* strains, including antibiotic resistant. Synergy in antibacterial activity was described for all the combinations and against all the *E. coli* strains tested.

## 3. Materials and Methods

### 3.1. Bacteria

Two bacterial strains from Spanish Type Culture Collection, *S. aureus* CECT 976 and *E. coli* CECT 434. These strains are used as model microorganisms for antimicrobial tests [9,11,55]. The strains prior stored at −80 °C were transferred to a Mueller–Hinton (Sigma-Aldrich, Lisbon, Portugal) agar plate at 37 °C. After an overnight growth, each strain was inoculated into MH broth at 37 °C and under agitation (150 rpm).

### 3.2. Antibiotics and NSAIDs

The antibiotics CIP, TET, KAN, STR were obtained from Sigma-Aldrich, and the solutions were prepared with water according to the Clinical and Laboratory Standards Institute (CLSI) guidelines [44]. The NSAIDs ASA, DCF and NPX were obtained from Sigma-Aldrich. PXC was obtained from Alfa Aesar. Solutions of PXC and ASA were prepared in dimethyl sulfoxide (DMSO), while DCF solutions were prepared in water for concentrations equal or below MIC. For concentrations above MIC, DCF solutions were prepared in DMSO. Due to the hydrophobic properties of PXC, it is mandatory the addition of lactose at 1% (w/v) to the MH broth, in order to stabilize the solution. Stock solutions of these compounds were stored at 4 °C for no longer than 24 h.

### 3.3. Antibacterial Susceptibility Tests

#### 3.3.1. Antibacterial Activity Assessment of Antibiotics by Disc Diffusion Method

Antibiotic susceptibility of *S. aureus* and *E. coli* was determined by disc diffusion method, according to Abreu et al. [9]. Overnight grown bacteria were adjusted to a 0.5 McFarland standard of. Bacterial cells were seeded in MH agar plates using a sterilized cotton swab. Then, a volume of 10 μL of CIP, TET, STR and KAN, prepared according to the CLSI guidelines [44], was added to 6 mm diameter sterile blank discs, which were placed on the agar plate. To facilitate the dissolution of CIP and TET, additional drops of HCl were added. Discs with water and the same amount of HCl used in antibiotics dissolution were used as negative controls.

Then, the plates were incubated for 24 h at 37 °C. After incubation, each inhibition zone diameter (IZD) was recorded. Three independent experiments were performed.

#### 3.3.2. Determination of the Minimum Inhibitory Concentration (MIC) and Minimum Bactericidal Concentration (MBC) of Selected Antibiotics and NSAIDs

The MIC of each compound was assessed by broth microdilution method in sterile 96-well microtiter plates (Orange Scientific, Braine-l’Alleud, Belgium), according to Oliveira et al. [32]. All of the NSAIDs and antibiotics were tested for both bacterial strains. Bacteria were inoculated into MH broth and the cultures were adjusted to an OD_600nm_ of 0.132 ± 0.02 (1 × 10^6^ CFU/mL) with fresh MH broth. Then, 180 µL of the cell culture and 20 µL of each compound were added to each well, performing final concentrations between 6.25 and 2000 µg/mL [56]. After 24 h incubation at 37 °C under agitation (150 rpm), MIC was defined as the lowest concentration of each compound that inhibited bacterial growth, based on absorbance measurements using a microplate reader (SPECTROstar Nano, BMG LABTECH, Ortenberg, Germany) at 600 nm (when final OD is equal or below the initial OD). MBC of each compound was also performed, transferring 10 µL of each concentration equal or above MIC (for each compound) to plate count agar (PCA) (Oxoid, UK) plates. After 24 h incubation at 37 °C, MBC was defined as the lowest concentration that totally inhibited bacterial growth [56]. At least three independent experiments were performed for each compound.

### 3.4. Dose–Response Curves

Dose–response curves were determined through the quantification of culturable cells, according to Borges et al. [57], with some modifications. Overnight bacterial cultures were adjusted to an OD_600nm_ of 0.132 ± 0.02 (1 × 10^6^ CFU/mL) and were added to a microcentrifuge tubes (Eppendorf, Hamburg, Germany), in a total volume of 1mL [56]. The microcentrifuge tubes with 900 µL and 100 µL of each NSAID were incubated for 1, 2, 3, 4, 5 and 6 h at 37 °C, under agitation (150 rpm). Cell suspensions without NSAIDs and with DMSO (10%, *v/v*) were used as negative controls. After exposure, 1:10 serial dilutions in saline solution (0.85%, NaCl) were performed, and 10 μL of each dilution was plated on plate count agar (PCA) plates and was incubated at 37 °C, during 24 h.

Colony forming units (CFU) were counted after the incubation period, and the results were expressed in log CFU/mL. At least three independent experiments were performed for each condition. Logarithmic reduction values were calculated for t = 6 h, according to Equation (1):

(1) $$\log\mathrm{reduction}=\log\mathrm{CFU}/{\mathrm{mL}}_{C}-\log\mathrm{CFU}/{\mathrm{mL}}_{\mathrm{NSAID}},$$

where C is the control. NSAIDs dissolved in DMSO have cells with DMSO as control, and NSAIDs dissolved in water have only cells as control.

### 3.5. Biofilm Control

#### 3.5.1. Biofilm Formation

Biofilm formation was performed according to Stepanovic et al. [58] with some modifications in order to assess the ability of NSAIDs to control 24 h old biofilms. Briefly, overnight cell cultures were adjusted to an OD_620nm_ of 0.04 ± 0.02 (1 × 10^8^ CFU/mL) with fresh MH broth, and 200 μL of cell suspensions was added to 96-well polystyrene microtiter plates [59]. The plates were incubated for 24 h at 37 °C under agitation (150 rpm).

#### 3.5.2. Exposure to NSAIDs

After biofilm formation, the content of the wells was caught up and the wells were washed with 200 μL of saline solution (0.85% NaCl) [32]. Afterwards, 20 μL of different NSAIDs in three different concentrations (MIC, 5× MIC and 10× MIC) and 180 μL of fresh MH broth were added to the wells, and the plates were incubated for 24 h at 37 °C under agitation (150 rpm). Wells only with cell suspensions and wells with DMSO (10%, *v/v*) were used as negative controls. Then, biofilms were analyzed in terms of biomass reduction, metabolic activity and culturability.

#### 3.5.3. Biofilm Control Analysis

##### Mass Quantification by Crystal Violet Staining

Mass quantification was performed according to Baptista et al. [11] in order to assess the biofilm mass reduction promoted by the selected NSAIDs. After NSAID exposure, the content of wells was pipetted, and the wells were washed with 200 μL saline solution (0.85%, NaCl) to remove all non-adherent and weakly adherent bacterial cells. Next, cells were fixed with 250 μL of 99% ethanol for 15 min, and the content was discarded at the end. Then, bacteria were stained with 1% crystal violet (CV; Sigma-Aldrich, Lisbon, Portugal) for 5 min, and the excess of stain was removed at the end. Finally, the stain was resolubilized with 200 μL of 33% (*v/v*) glacial acetic acid, and OD was measured in a microplate reader (SpectraMax M2e) at 570 nm. At least three independent experiments were performed. The results were expressed as the biofilm removal (%) regarding biofilms non-exposed to NSAIDs (Equation (2)):

(2) $$\%BR=\frac{OD_{C}-OD_{W}}{OD_{C}}\times 100,$$

where *%BR* is the percentage of biofilm removal, *OD_C_* is the *OD*_570nm_ value of the control wells and *OD_W_* is the *OD*_570nm_ value for the NSAID-treated wells.

##### Metabolic Activity Quantification by Alamar Blue Assay

Metabolic activity quantification was performed according to Baptista et al. [11] in order to assess the metabolic inactivation of the biofilm cells promoted by the selected NSAIDs. After NSAID exposure, wells were pipetted and washed as mentioned for CV staining. For the staining procedure, 10 μL of alamar blue solution (Sigma-Aldrich, Lisbon, Portugal) and 190 μL of fresh MH broth were added to each well. Then, the microtiter plates were incubated for 20 min in the dark at 37 °C under agitation (150 rpm).

Finally, fluorescence intensity was measured at excitation and emission wavelengths of 570 nm and 590 nm, respectively, using a microplate reader (FLUOstar Omega) (BMG LABTECH, Ortenberg, Germany). At least three independent experiments were performed. The results are expressed as the biofilm inactivation (%) regarding biofilms non-exposed to NSAIDs (Equation (3)):

(3) $$\%BI=\frac{FI_{C}-FI_{W}}{FI_{C}}\times 100,$$

where *%BI* is the percentage of biofilm inactivation, *FI_C_* is the fluorescence intensity of control wells and *FI_W_* is the fluorescence intensity for the NSAID-treated wells.

##### Biofilm Culturable Cells Quantification by Plate Count Method

Biofilm culturable cell quantification was performed according to Baptista et al. [11] in order to assess the culturability reduction of biofilm cells promoted by selected NSAIDs. After the treatment with NSAIDs, the content of the wells was removed, and the wells were washed to remove non-adherent bacteria, as mentioned before for CV and alamar blue staining. Afterwards, the attached cells were scraped three times with 200 μL of saline solution (0.85%, NaCl) for 1 min each.

The content of each well (600 μL) was transferred to a microcentrifuge tubes (Eppendorf, Hamburg, Germany), and it was also added to the 400 μL saline solution. Then, 1:10 serial dilutions were performed in saline solution, and 10 μL of each dilution was plated on PCA plates. Plates were incubated at 37 °C during 24 h. At least three independent experiments were performed.

CFUs were counted (10 < CFU < 200) after the incubation period, and the results were expressed per square centimeter of microtiter plate well—CFU/cm^2^, according to Equations (4) and (5).

(4) $$\frac{\mathrm{CFU}}{\mathrm{mL}}=\frac{N}{\mathrm{SV}\times \mathrm{Dilution}},$$

where N is the number of CFU in the PCA plates and SV is the volume of the sample in mL.

(5) $$\frac{\mathrm{CFU}}{{\mathrm{cm}}^{2}}=\frac{\frac{\mathrm{CFU}}{\mathrm{mL}}\times WV}{1.53},$$

where *WV* is the well’s volume (0.2 mL) and 1.53 is the area of the well in cm^2^. The final results were expressed in log CFU/cm^2^.

#### 3.5.4. Biofilm Control Activity Classification

The effects of NSAIDs on biofilm control were classified according to the following conditions proposed by Lemos et al. [60], where “I” indicates inactivation and “R” indicates removal:

- Low efficacy: I or R < 25%;
- Moderate efficacy: 25% ≤ I or R < 60%;
- High efficacy: 60% ≤ I or R < 90%;
- Excellent efficacy: 90% ≤ I or R ≤ 100%.

#### 3.5.5. Dual Combinations of NSAIDs with Antibiotics

In order to evaluate the combined effect of NSAIDs and antibiotics, an overnight culture was standardized to an OD_600nm_ of 0.04 ± 0.02 with fresh MH broth. Biofilms were formed in a 96-well microtiter plate as previously stated in Section 3.5.1. Afterwards, 10 μL of each NSAID, 10 μL of each antibiotic and 180 mL of fresh MHB were added to the microplates, which were incubated at 37 °C for 24 h. Then, the biofilms were analyzed in terms of reduction of biomass, metabolic activity and culturability, as described in the previously.

Subsequently, in order to classify the interaction between NSAIDs and antibiotics when com- pared to NSAIDs and antibiotics alone, the total combinatorial index (∑*C_I_*) for each combination was calculated and scored according to the score proposed by Baptista et al. [11]. The combinatorial index of NSAIDs (*C_I_*_|*N*_) was determined through Equation (6):

(6) $$C_{I|N}=\frac{R_{N}}{R_{N|A}},$$

where *R_N_* are the results obtained for NSAIDs alone (at MIC, 5× MIC and 10× MIC) for each method (biofilm removal, biofilm inactivation and log CFU/cm^2^ reduction); *R_N|A_* are the results obtained for the combination of NSAIDs (at MIC, 5× MIC and 10× MIC) with antibiotics (at MIC) for each method. The combinatorial index of antibiotics (*C_I|A_*) was determined in a similar way, according to Baptista et al. [11]. Afterwards, ∑*C_I_* was calculated by Equation (7):

(7) $$\Sigma C_{I}=C_{I|N}+C_{I|A},$$

∑*C_I_* values were then scored and classified according to the interactions between NSAIDs and antibiotics in *S. aureus* and *E. coli* biofilms:

- <0.5—synergistic (+++);
- 0.5 to 2—additive (++);
- 2 to 4—indifferent (+);
- >4—antagonistic (–).

### 3.6. Statistical Analysis

Mean and standard deviation (SD) values were determined for all performed tests. All the tests were performed at least three times and with several replicates. The data were analyzed using t-student test from Microsoft Excel 2018. One-way ANOVA was also performed assuming a normal distribution. Statistical analysis was determined assuming a confidence level ≥95%. Thus, *p* < 0.05 was considered statistically significant.

## 4. Conclusions

This study demonstrated the antibacterial activity of selected NSAIDs against *E. coli* and *S. aureus*. Among the selected NSAIDs, NPX was the only NSAID which did not demonstrate antibacterial activity in the range of concentrations tested (up to 2000 μg/mL). On the other hand, PXC showed antibacterial activity against *E. coli*, and DCF showed antibacterial activity against *S. aureus*. ASA was the only NSAID which demonstrated broad-spectrum activity, with antibacterial activity against both bacteria. Moreover, *S. aureus* appeared to be more resistant to ASA than *E. coli*. Bactericidal activities were not found for any of the NSAIDs in the range of concentrations tested. NSAIDs also demonstrated their ability to control preformed biofilms essentially by metabolic inactivation and CFU reduction. In fact, the three NSAIDs showed high efficacy in the reduction of the metabolic activity of *E. coli* and *S. aureus* biofilms (>60%). Furthermore, DCF promoted total log CFU/cm^2^ reduction of *S. aureus* biofilm cells, and ASA promoted total log CFU/cm^2^ reductions of both *E. coli* and *S. aureus* biofilms. PXC was the only NSAID that demonstrated ability to remove biofilm cells. The present study also highlights NSAIDs as an alternative to the use of antibiotics. In fact, in most of the cases, the antibiofilm activity of NSAIDs was similar or even better than the antibiofilm activity of KAN or TET in terms of both metabolic activity and CFU/cm^2^ reduction. Furthermore, in most of the cases, NSAIDs combined with KAN or TET promoted similar or worst results than NSAIDs or antibiotics used alone. In addition, no synergistic interactions were found between the selected NSAIDs and antibiotics. In fact, NSAIDs presented additive interactions with KAN or TET in most of the combinations. In conclusion, repurposing of some NSAIDs for antimicrobial purposes seems to be a promising therapeutic strategy—besides the antibacterial and antibiofilm activity, the ADME properties and their effects on the human organism are well known. However, careful management in therapeutic human use is required, because the plasma concentration of the selected NSAIDs after administration is below that used in this study to inhibit bacterial growth [61,62,63]. On the other hand, topical formulations of NSAIDs in current commercial use can contain levels between 0.5 and 10% (*w/w*): DCF is used in a range of 1 and 3% [64], PXC is used in a range of 0.5 and 1% [65,66] and NPX is used in a range of 5 and 10% [67]. These concentrations are higher than those required to promote antimicrobial and antibiofilm activity in the present study. Therefore, NSAIDs can be used in a first instance as antiseptic and/or disinfectant in topical formulations.

## Figures and Tables

**Table 1:** MIC (minimum inhibitory concentration) and MBC (minimum bactericidal concentration) of PXC (piroxicam), DCF (diclofenac sodium), ASA (acetylsalicylic acid) and NPX (naproxen sodium) against *E. coli* CECT 343 and *S. aureus* CECT 976. Data are expressed as mean ± SD of three independent assays.

NSAIDs	Bacteria	MIC (μg/mL)
PXC	*E. coli*	800 ± 0 *
	*S. aureus*	>2000
DCF	*E. coli*	>2000
	*S. aureus*	2000 ± 0
ASA	*E. coli*	1750 ± 0
	*S. aureus*	2000 ± 0
NPX	*E. coli*	>2000
	*S. aureus*	>2000

* With lactose.

**Table 2:** IZD (Inhibition zone diameters), in mm, promoted by CIP (ciprofloxacin), TET (tetracycline), STR (streptomycin) and KAN (kanamycin). Data are expressed as mean ± SD of three independent assays. The two antibiotics with lowest IZD values per strain are presented in bold.

Bacterial Strain	Antibiotic	IZD (mm)
*E. coli* CECT 434	CIP_5μg/disc_	38.8 ± 3.1
	TET_30μg/disc_	26.5 ± 1.2
	STR_10μg/disc_	39.7 ± 3.3
	KAN_30μg/disc_	29.8 ± 2.1
*S. aureus* CECT 976	CIP_5μg/disc_	43.2 ± 2.1
	TET_30μg/disc_	37.3 ± 1.4
	STR_10μg/disc_	43.7 ± 0.0
	KAN_30μg/disc_	28.5 ± 0.2

**Table 3:** MIC and MBC of KAN and TET against *E. coli* CECT 343 and *S. aureus* CECT 976.

Antibiotic	MIC/MBC (μg/mL)	*E. coli*	*S. aureus*
KAN	MIC	24	3
	MBC	24	24
TET	MIC	6	4
	MBC	64	48

**Table 4:** ∑*C_I_* scoring of the selected NSAIDs (at MIC, 5× MIC and 10× MIC) with TET (tetracycline) and KAN (kanamycin) (at MIC) in terms of biomass removal, biofilm inactivation and culturability.

Bacterial Strain	NSAID/Antibiotic	Concentration (μg/mL)	Biofilm Removal	Biofilm Inactivation	Biofilm Culturability
*E. coli*	PXC + KAN	MIC + MIC	+	+	++
		5MIC + MIC	+	+	++
		10MIC + MIC	++	+	++
	PXC + TET	MIC + MIC	-	++	++
		5MIC + MIC	+	++	++
		10MIC + MIC	++	++	++
	ASA + KAN	MIC + MIC	++	++	+
		5MIC + MIC	++	++	++
		10MIC + MIC	++	++	++
	ASA + TET	MIC + MIC	++	++	++
		5MIC + MIC	++	++	++
		10MIC + MIC	++	++	++
*S. aureus*	DCF + KAN	MIC + MIC	+	++	+
		5MIC + MIC	++	++	+
		10MIC + MIC	++	++	++
	DCF + TET	MIC + MIC	++	++	++
		5MIC + MIC	++	++	+
		10MIC + MIC	++	++	++
	ASA + KAN	MIC + MIC	++	++	+
		5MIC + MIC	++	++	++
		10MIC + MIC	++	++	++
	ASA + TET	MIC + MIC	++	++	++
		5MIC + MIC	++	++	++
		10MIC + MIC	++	++	++

(++)—Additive; (+)—Indifferent; (-)—Antagonistic.

## Supplementary Materials

The following are available online at https://www.mdpi.com/2079-6382/9/9/591/s1, Table S1: log CFU/mL reduction values for NSAIDs at 1/2× MIC, MIC and a concentration above MIC, after 6 h. Table S2: Percentages of mass reduction and inactivation and log CFU/cm^2^ reduction of *E. coli* and *S. aureus* biofilms grown in the presence of the selected NSAIDs at different concentrations (MIC, 5× MIC and 10× MIC). Table S3: Percentages of mass reduction inactivation and log CFU/cm^2^ reduction of *E. coli* and *S. aureus* biofilms grown in the presence of KAN and TET at MIC. Table S4: Percentages of mass reduction and cell inactivation and log CFU/cm^2^ reduction of *E. coli* and *S. aureus* biofilms grown in the presence of the selected NSAIDs at different concentrations and antibiotics at MIC. Table S5: Classification of the efficacy of the selected NSAIDs (at MIC, 5× MIC and 10× MIC) and KAN and TET (at MIC) in the control of *E. coli* and *S. aureus* biofilms in terms of biofilm mass reduction and inactivation.
