# Supplementary material for: NSAIDs as a Drug Repurposing Strategy for Biofilm Control
Source: Antibiotics (Basel). 2020 Sep 10;9(9):591. doi: 10.3390/antibiotics9090591 (PMC7558876; doi:10.3390/antibiotics9090591)
Supplement: Supplementary file 1 [file antibiotics-09-00591-s001.pdf]

Supplementary Tables

1. **Table S1.** Log CFU/mL reduction values for NSAIDs at 1/2×MIC, MIC and a concentration above MIC, after 6h
2. **Table S2.** Percentage of biomass reduction and cell inactivation and log CFU/cm^2 reduction of *E. coli* and *S. aureus* biofilms, grown in the presence of the selected NSAIDs at different concentrations (MIC, 5×MIC and 10×MIC). Mean values ± Standard deviation are illustrated
3. **Table S3.** Percentage of biomass reduction and cell inactivation and log CFU/cm^2 reduction of *E. coli* and *S. aureus* biofilms, grown in the presence of KAN and TET at MIC. Mean values ± Standard deviation are illustrated
4. **Table S4.** Percentage of biomass reduction and cell inactivation and log CFU/cm^2 reduction of *E. coli* and *S. aureus* biofilms, grown in the presence of the selected NSAIDs at different concentrations and antibiotics at MIC. Mean values ± Standard deviation are illustrated
5. **Table S5.** Classification of the efficacy of the selected NSAIDs (at MIC, 5×MIC and 10×MIC) and KAN and TET (at MIC), in the control of *E. coli* and *S. aureus* biofilms, in terms of biomass reduction and biofilm inactivation

Table S1. Log CFU/mL reduction values for NSAIDs at 1/2×MIC, MIC and a concentration above MIC, after 6h.

E. coli			***S. aureus***		
**NSAID**	**C (μg/mL)**	**log reduction**	**NSAID**	**C (μg/mL)**	**log reduction**
PXC	400	1.20	DCF	1000	2.50
	800	1.27		2000	3.86
	1500	1.34		2500	5.68
ASA	875	0.19	ASA	1000	0.05
	1750	0.83		2000	0.92
	2500	0.90		2500	1.06

Table S2. Percentage of biomass reduction and cell inactivation and log CFU/cm^2 reduction of *E. coli* and *S. aureus* biofilms, grown in the presence of the selected NSAIDs at different concentrations (MIC, 5×MIC and 10×MIC).

**Biomass reduction (%)**	**NSAIDs**			
	E. coli		*S. aureus*	
	PXC	ASA	DCF	ASA
MIC	28.6 ± 2.7	-	-	-
5×MIC	16.8 ± 0.1	-	-	-
10×MIC	-	-	-	-
Biofilm inactivation (%)	NSAIDs			
	E. coli		*S. aureus*	
	PXC	ASA	DCF	ASA
MIC	73.2 ± 2.4	74.3 ± 3.8	80.4 ± 2.2	82.7 ± 2.4
5×MIC	72.9 ± 2.2	74.1 ± 4.9	86.6 ± 3.2	84.0 ± 2.7
10×MIC	66.1 ± 3.5	74.1 ± 4.4	86.7 ± 3.3	83.2 ± 3.8
log CFU/cm^2 reduction	NSAIDs			
	E. coli		*S. aureus*	
	PXC	ASA	DCF	ASA
Negative control (DMSO)	0.68 ± 0.47		0.22 ± 0.40	
MIC	3.24 ± 0.48	3.55 ± 0.47	2.22 ± 1.08	3.33 ± 0.32
5×MIC	3.58 ± 0.47	6.46 ± 0.47	3.47 ± 0.67	6.19 ± 0.31
10×MIC	3.78 ± 0.49	6.46 ± 0.47	6.19 ± 0.31	6.19 ± 0.31

Mean values ± Standard deviation are illustrated.

Table S3. Percentage of biomass reduction and cell inactivation and log CFU/cm^2 reduction of *E. coli* and *S. aureus* biofilms, grown in the presence of KAN and TET at MIC.

**Biomass reduction (%)**	**Antibiotics**			
	E. coli		*S. aureus*	
	KAN	TET	KAN	TET
MIC	22.8 ± 1.8	21.5 ± 1.3	30.6 ± 1.0	31.2 ± 2.2
Biofilm inactivation (%)	Antibiotics			
	E. coli		*S. aureus*	
	KAN	TET	KAN	TET
MIC	80.8 ± 2.2	66.8 ± 1.0	43.5 ± 2.8	26.8 ± 1.1
log CFU/cm^2 reduction	Antibiotics			
	E. coli		*S. aureus*	
	KAN	TET	KAN	TET
MIC	3.72 ± 0.37	2.21 ± 0.06	1.37 ± 0.33	0.85 ± 0.46

Mean values ± Standard deviation are illustrated.

Table S4. Percentage of biomass reduction and cell inactivation and log CFU/cm^2 reduction of *E. coli* and *S. aureus* biofilms, grown in the presence of the selected NSAIDs at different concentrations and antibiotics at MIC.

**Biomass reduction (%)**	**NSAIDs + KAN**				**NSAIDs + TET**			
	E. coli		*S. aureus*		*E. coli*		*S. aureus*	
	PXC	ASA	DCF	ASA	PXC	ASA	DCF	ASA
MIC	17.8 ± 0.9	30.0 ± 1.1	12.2 ± 0.6	31.4 ± 1.3	12.4 ± 0.2	25.4 ± 0.1	16.2 ± 1.1	28.4 ± 1.5
5×MIC	19.0 ± 1.1	30.0 ± 0.3	16.8 ± 2.0	28.1 ± 0.5	16.3 ± 0.7	20.1 ± 1.9	22.5 ± 0.4	27.4 ± 0.4
10×MIC	25.2 ± 2.0	21.7 ± 0.8	23.5 ± 1.9	16.7 ± 2.0	20.5 ± 1.3	12.1 ± 1.6	32.4 ± 0.8	22.2 ± 0.4
Biofilm inactivation (%)	NSAIDs + KAN				NSAIDs + TET			
	E. coli		*S. aureus*		*E. coli*		*S. aureus*	
	PXC	ASA	DCF	ASA	PXC	ASA	DCF	ASA
MIC	63.0 ± 0.5	86.3 ± 3.6	82.6 ± 0.5	73.3 ± 1.6	70.3 ± 0.5	88.8 ± 1.9	80.2 ± 6.7	74.4 ± 2.2
5×MIC	67.2 ± 0.0	92.6 ± 1.1	89.3 ± 4.1	76.2 ± 2.6	74.0 ± 1.0	92.7 ± 1.1	89.2 ± 4.7	76.2 ± 1.6
10×MIC	70.0 ± 1.4	92.5 ± 1.3	89.2 ± 3.7	77.2 ± 1.6	85.3 ± 0.8	92.7 ± 1.4	89.2 ± 4.7	76.7 ± 2.0
log CFU/cm^2 reduction	NSAIDs + KAN				NSAIDs + TET			
	E. coli		*S. aureus*		*E. coli*		*S. aureus*	
	PXC	ASA	DCF	ASA	PXC	ASA	DCF	ASA
Negative control (DMSO)	0.68 ± 0.47		0.22 ± 0.40		0.68 ± 0.47		0.22 ± 0.40	
MIC	2.48 ± 0.53	4.04 ± 0.68	3.00 ± 0.31	2.73 ± 0.70	1.76 ± 0.47	1.35 ± 0.84	0.99 ± 0.33	1.04 ± 0.31
5×MIC	2.57 ± 0.55	6.46 ± 0.47	3.15 ± 0.32	6.19 ± 0.31	1.96 ± 0.47	3.86 ± 0.49	3.05 ± 0.33	2.06 ± 0.31
10×MIC	2.64 ± 0.56	6.46 ± 0.47	6.19 ± 0.31	6.19 ± 0.31	2.24 ± 0.47	6.46 ± 0.47	6.19 ± 0.31	6.19 ± 0.31

Mean values ± Standard deviation are illustrated.

Table S5. Classification of the efficacy of the selected NSAIDs (at MIC, 5×MIC and 10×MIC) and KAN and TET (at MIC), in the control of *E. coli* and *S. aureus* biofilms, in terms of biomass reduction and biofilm inactivation.

**Bacteria**	**Antibiotic**	**MIC**	**Biomass reduction**	**Biofilm inactivation**
E. coli	KAN	MIC	Low efficacy	High efficacy
	TET	MIC	Low efficacy	High efficacy
	PXC	MIC	Moderate efficacy	High efficacy
		5× MIC	Low efficacy	High efficacy
		10× MIC	Low efficacy	High efficacy
	PXC+KAN	MIC + MIC	Low efficacy	High efficacy
		5× MIC + MIC	Low efficacy	High efficacy
		10× MIC + MIC	Moderate efficacy	High efficacy
	PXC+TET	MIC + MIC	Low efficacy	High efficacy
		5×MIC + MIC	Low efficacy	High efficacy

		10×MIC + MIC	Low efficacy	High efficacy
	ASA	MIC	Low efficacy	High efficacy
		5×MIC	Low efficacy	High efficacy
		10×MIC	Low efficacy	High efficacy
	ASA+KAN	MIC + MIC	Moderate efficacy	High efficacy
		5×MIC + MIC	Moderate efficacy	Excellent efficacy
		10×MIC + MIC	Low efficacy	Excellent efficacy
	ASA+TET	MIC + MIC	Moderate efficacy	High efficacy
		5×MIC + MIC	Low efficacy	Excellent efficacy
		10×MIC + MIC	Low efficacy	Excellent efficacy
S. aureus	KAN	MIC	Moderate efficacy	Moderate efficacy
	TET	MIC	Moderate efficacy	Moderate efficacy
	DCF	MIC	Low efficacy	High efficacy
		5×MIC	Low efficacy	High efficacy
		10×MIC	Low efficacy	High efficacy
	DCF+KAN	MIC + MIC	Low efficacy	High efficacy
		5×MIC + MIC	Low efficacy	High efficacy
		10×MIC + MIC	Low efficacy	High efficacy
	DCF+TET	MIC + MIC	Low efficacy	High efficacy
		5×MIC + MIC	Low efficacy	High efficacy
		10×MIC + MIC	Moderate efficacy	High efficacy
	ASA	MIC	Low efficacy	High efficacy
		5×MIC	Low efficacy	High efficacy
		10×MIC	Low efficacy	High efficacy
	ASA+KAN	MIC + MIC	Moderate efficacy	High efficacy
		5×MIC + MIC	Moderate efficacy	High efficacy
		10×MIC + MIC	Low efficacy	High efficacy
	ASA+TET	MIC + MIC	Moderate efficacy	High efficacy
		5×MIC + MIC	Moderate efficacy	High efficacy
		10×MIC + MIC	Low efficacy	High efficacy
